# Supplementary material for: Applicability of Obesity-Related SNPs and Their Effect Size Measures Defined on Populations with European Ancestry for Genetic Risk Estimation among Roma
Source: Genes (Basel). 2020 May 6;11(5):516. doi: 10.3390/genes11050516 (PMC7720118; doi:10.3390/genes11050516)
Supplement: Supplementary file 1 [file genes-11-00516-s001.pdf]

Overview of the present results

Among both populations, these *FTO* SNPs likely include the functional variant that may explain the risk and associated phenotype, known as the causal variant. In the literature, *FTO* SNPs are also the most significantly associated with BMI and highly correlated (LD: r^2 >0.90) in Asian and European populations (see **Figure S4 below)**[1].

These results are in line with data reported in a study on Spanish Roma, aiming at defining the association between previously GWAS-identified genetic variants predisposing to obesity–related phenotypes in European subjects. This research reported SNPs significantly associated with body fat accumulation and distribution in or near six genes (*BDNF*, *FAIM2*, *FTO*, *MC4R*, *NEGR1* and *SH2B1*) [2].

In our analysis, *FTO* gene variants, that may affect food intake variation (but not energy expenditure) [3-5], were found to have a directionally consistent association with obesity-related phenotypes in both populations. Murine models have confirmed that *FTO* may modulate functions related to energy balance [6, 7] and may influence pathways that regulate protein intake [8-10]. However, recent work suggests that the genetic variants of the *FTO* gene may exert their effects via neighbouring genes at the locus [11]. Concerning Roma population, a study conducted in Slovakian Roma found a significant association of rs9939609 (*FTO*) with obesity [12]. However, the same variant did not show significant association with obesity-related phenotypes in Spanish Roma population [2].

Our analysis identified nine out of the twenty preselected SNPs with no significant association with any obesity- or WC-related phenotype in neither of the populations studied. Two SNPs (rs2867125 and rs6548238) in the *TMEM18* gene showed no association with WC defined by using the ATPIII criteria in both populations, but a significant association with WC was found in the HG population, but not in HR if IDF classification criteria were applied. These SNPs have been previously reported (in European and African populations) to be associated with obesity (BMI and weight) [13] and it was reported (in European population) to be involved in adult and childhood obesity as well as type 2 diabetes [14].

Another variant that was found to have a significant effect on obesity and WC, by ATPIII criteria, was the rs12970134 in the *MC4R* gene. This SNP has been described in the literature and it is associated with appetite and total energy intake regulation, consumption of fat, protein and carbohydrates [15, 16]. Mutations in *MC4R* account for up to 5% of extreme early-onset obesity and are the most prevalent genetic cause, together with *FTO* variants [17]. Concerning the protective effect of genetic variants, the rs16139 SNP in the *NPY* gene showed a protective effect in HG subjects. *NPY* has potent orexigenic effects as a neuropeptide [18]and interacts with leptin to regulate food intake[19, 20]. Because of the Asian origin of Roma and the European origin of Hungarian general population, results meeting IDF_{ASIA} criteria for the Roma and IDF_{EURO} for the Hungarian general population can be considered as most probable. Our findings in case of the rs9944349 SNP showed the opposite association, which can only be partially explained by the above described finding, namely the IBD segment length shared by Roma and the Central European populations was higher than the others from Asian populations. This fact could at least partly explain the association of rs9944349 with WC in Roma according the IDF_{EURO} criteria, but we have no explanation based on the origin of the HG population regarding the association with the IDF_{ASIA} criteria in case of the HG population. However, probability values in case of the rs9941349 showed only borderline significance (*see Table S5 below*) and the effect of SNPs on WC according to neither the IDF_{EURO} nor the IDF_{ASIA} criteria did not differ significantly between the study populations (*Figure 1 in the manuscript*).

Table S3: The results of adjusted (by age and sex) linear and logistic regression models of BMI in Hungarian general and Roma populations

SNP	Gene	Risk allele	BMI (lin. reg.)							BMI categorical (BMI≤25 vs. BMI≥30; log. reg.)						
			HG (N=1496)			HR (N=1141)			p-value for the comparative analysis‡	HG (N=580)			HR (N=452)			p-value for the comparative analysis‡
			Beta	Std. Err.	p-value	Beta	Std. Err.	p-value		OR	[95% CI]	p-value	OR	[95% CI]	p-value	
rs10938397	*GNPDA2*	G	0.299	0.212	0.160	0.619	0.359	0.085	0.485	1.133	0.927 - 1.387	0.223	1.210	0.983 - 1.490	0.072	0.694
rs1121980	*FTO*	A	0.553	0.205	0.007	0.604	0.356	0.090	0.888	1.273	1.045 - 1.550	0.016	1.292	1.050 - 1.590	0.015	0.971
rs1137101	*LEPR*	G	0.099	0.212	0.639	-0.106	0.356	0.767	0.630	1.048	0.853 - 1.287	0.657	0.901	0.733 - 1.109	0.326	0.310
rs12970134	*MC4R*	A	0.368	0.240	0.126	0.066	0.421	0.875	0.576	1.273	1.008 - 1.607	0.043	1.071	0.843 - 1.360	0.574	0.317
rs1501299	*ADIPOQ*	T	0.049	0.231	0.832	0.354	0.387	0.360	0.499	1.121	0.898 - 1.399	0.314	1.051	0.840 - 1.314	0.665	0.675
rs1558902	*FTO*	A	0.482	0.205	0.019	0.628	0.360	0.081	0.697	1.247	1.025 - 1.517	0.028	1.263	1.025 - 1.554	0.028	0.950
rs16139	*NPY*	C	-0.602	0.517	0.244	-0.686	1.186	0.563	0.955	0.609	0.377 - 0.985	0.043	0.690	0.333 - 1.427	0.316	0.763
rs17782313	*MC4R*	C	0.320	0.247	0.194	0.179	0.422	0.672	0.825	1.244	0.978 - 1.582	0.075	1.132	0.890 - 1.441	0.312	0.605
rs1801282	*PPARγ*	C	-0.005	0.314	0.988	-1.188	0.807	0.141	0.177	1.226	0.901 - 1.669	0.194	0.778	0.488 - 1.241	0.292	0.127
rs2241766	*ADIPOQ*	G	-0.152	0.337	0.653	-0.473	0.553	0.392	0.643	0.943	0.684 - 1.299	0.719	0.999	0.730 -1.368	0.996	0.757
rs2815752	*NEGR1*	C	0.289	0.222	0.193	-0.088	0.396	0.824	0.383	1.208	0.975 - 1.497	0.084	1.071	0.849 - 1.351	0.565	0.436
rs2867125	*TMEM18*	C	0.251	0.266	0.345	0.015	0.494	0.975	0.799	1.149	0.889 - 1.484	0.288	0.975	0.731 -1.301	0.865	0.454
rs6265	*BDNF*	C	0.434	0.262	0.097	0.167	0.551	0.761	0.567	1.256	0.974 - 1.620	0.079	1.020	0.738 - 1.409	0.904	0.312
rs6499640	*FTO*	A	0.226	0.209	0.279	0.380	0.351	0.279	0.751	1.078	0.881 - 1.318	0.466	1.087	0.885 - 1.333	0.427	0.995
rs6548238	*TMEM18*	C	0.308	0.265	0.245	0.294	0.539	0.585	0.900	1.150	0.890 - 1.485	0.284	1.117	0.813 - 1.536	0.495	0.964
rs659366	*UCP2*	C	-0.049	0.219	0.821	0.402	0.369	0.277	0.378	0.997	0.809 - 1.228	0.975	1.074	0.868 - 1.330	0.511	0.649
rs660339	*UCP2*	G	-0.100	0.217	0.645	0.295	0.360	0.413	0.422	0.952	0.773 - 1.172	0.642	1.064	0.862 - 1.313	0.566	0.475
rs925946	*BDNF*	T	0.316	0.243	0.194	0.157	0.362	0.665	0.742	1.120	0.891 - 1.407	0.331	1.112	0.903 - 1.368	0.317	0.994
rs9939609	*FTO*	A	0.515	0.205	0.012	0.756	0.357	0.035	0.525	1.273	1.045 - 1.550	0.016	1.336	1.085 - 1.646	0.006	0.758
rs9941349	*FTO*	T	0.618	0.206	0.003	0.770	0.357	0.031	0.666	1.324	1.085 - 1.615	0.006	1.312	1.065 - 1.616	0.011	0.922

‡ Difference in association with quantitative traits between study populations. *p≤0.05 and **p≤0.0025.
Results in grey highlights the significant associations between the SNP and BMI by populations.

Table S4: The results of adjusted (by age and sex) linear and logistic regression models of waist circumference in Hungarian general and Roma populations

			Waist							Waist categorical (Females: WC≥88cm vs. WC<88 mc (reference), Males: WC≥102cm vs. WC<102 cm (reference))						
SNP	Gene	Risk allele	HG (N=1496)			HR (N=1141)			p-value‡	HG (N=1496)			HR (N=1141)			p-value‡
			Beta	Std. Err.	p-value	Beta	Std. Err.	p-value		OR	[95% CI]	p-value	OR	[95% CI]	p-value	
rs10938397	*GNPDA2*	G	0.655	0.489	0.181	1.222	0.757	0.107	0.486	1.155	0.981 - 1.360	0.085	1.202	1.000 - 1.446	0.051	0.720
rs1121980	*FTO*	A	1.516	0.472	0.001	1.682	0.752	0.025	0.679	1.120	0.956 - 1.314	0.161	1.210	1.006 - 1.454	0.043	0.594
rs1137101	*LEPR*	G	0.320	0.488	0.512	0.316	0.749	0.673	0.925	1.021	0.867 - 1.203	0.802	1.038	0.865 - 1.246	0.690	0.935
rs12970134	*MC4R*	A	1.007	0.553	0.069	-0.543	0.888	0.541	0.143	1.195	0.991 - 1.441	0.062	1.011	0.814 - 1.255	0.924	0.233
rs1501299	*ADIPOQ*	T	0.124	0.532	0.816	-0.231	0.817	0.777	0.738	1.038	0.868 - 1.242	0.680	0.932	0.762 - 1.140	0.494	0.414
rs1558902	*FTO*	A	1.318	0.472	0.005	1.513	0.758	0.046	0.730	1.107	0.945 - 1.298	0.208	1.195	0.993 - 1.439	0.059	0.560
rs16139	*NPY*	C	-1.883	1.190	0.114	-1.014	2.559	0.692	0.727	0.703	0.470 - 1.052	0.087	0.757	0.400 - 1.432	0.391	0.853
rs17782313	*MC4R*	C	0.836	0.569	0.142	-0.470	0.892	0.598	0.215	1.155	0.954 - 1.400	0.140	1.025	0.824 - 1.274	0.826	0.391
rs1801282	*PPARγ*	C	0.340	0.724	0.638	-3.507	1.699	0.039	0.029*	1.132	0.887 - 1.443	0.319	0.733	0.488 - 1.101	0.135	0.068
rs2241766	*ADIPOQ*	G	-0.704	0.777	0.365	-1.301	1.163	0.264	0.693	0.860	0.883 - 1.213	0.261	0.905	0.681 - 1.204	0.495	0.854
rs2815752	*NEGR1*	C	0.604	0.511	0.237	0.793	0.835	0.342	0.690	1.123	0.947 - 1.332	0.182	1.295	1.053 - 1.592	0.014	0.324
rs2867125	*TMEM18*	C	0.318	0.612	0.604	-0.267	1.042	0.798	0.791	1.032	0.840 - 1.267	0.767	0.939	0.729 - 1.208	0.624	0.370
rs6265	*BDNF*	C	0.766	0.603	0.204	0.171	1.161	0.883	0.443	1.255	1.024 - 1.538	0.028	0.999	0.753 - 1.325	0.994	0.352
rs6499640	*FTO*	A	0.607	0.481	0.206	1.192	0.739	0.107	0.563	1.014	0.863 - 1.192	0.862	1.056	0.882 - 1.264	0.556	0.647
rs6548238	*TMEM18*	C	0.354	0.610	0.562	-0.158	1.138	0.890	0.905	1.040	0.848 - 1.276	0.705	1.012	0.767 - 1.335	0.933	0.622
rs659366	*UCP2*	C	-0.146	0.501	0.770	0.949	0.779	0.224	0.394	0.936	0.792 - 1.107	0.441	1.009	0.836 - 1.218	0.924	0.334
rs660339	*UCP2*	G	-0.368	0.501	0.462	0.806	0.760	0.289	0.305	0.906	0.767 - 1.071	0.248	0.989	0.822 - 1.190	0.906	0.331
rs925946	*BDNF*	T	0.606	0.560	0.279	-0.162	0.764	0.832	0.374	1.192	0.988 - 1.438	0.067	1.070	0.888 - 1.290	0.475	0.452
rs9939609	*FTO*	A	1.333	0.472	0.005	1.596	0.754	0.035	0.618	1.086	0.926 - 1.273	0.309	1.201	0.998 - 1.444	0.053	0.475
rs9941349	*FTO*	T	1.567	0.474	0.001	1.546	0.754	0.040	0.801	1.114	0.950 - 1.307	0.184	1.245	1.034 - 1.498	0.021	0.448

‡Difference in association with quantitative traits between study populations. *p≤0.05 and **p≤0.0025.

Results in grey highlights the significant associations between the SNP and BMI by populations.

Table S5: The results of adjusted (by age and sex) linear and logistic regression models of waist circumference according to IDF European and Asian criteria in Hungarian general and Roma populations

			Waist circumference (European; Females: WC≥80cm vs. WC<80 mc (ref.), Males: WC≥94cm vs. WC<94 cm (ref.))							Waist categorical (Asian ; Females: WC≥80cm vs. WC<80 cm (ref.), Males: WC≥90cm vs. WC<90cm (ref.))						
SNP	Gene	Risk allele	HG (N=1496)			HR (N=1141)			p-value‡	HG (N=1496)			HR (N=1141)			p-value‡
			OR	[95% CI]	p-value	OR	[95% CI]	p-value		OR	[95% CI]	p-value	OR	[95% CI]	p-value	
rs10938397	*GNPDA2*	G	1.046	0.880 - 1.243	0.610	1.147	0.955 - 1.379	0.143	0.474	0.973	0.812 - 1.167	0.770	1.159	0.963 - 1.394	0.119	0.189
rs1121980	*FTO*	A	1.137	0.960 - 1.346	0.136	1.182	0.985 - 1.419	0.072	0.764	1.169	0.979 - 1.397	0.084	1.154	0.961 - 1.385	0.125	0.918
rs1137101	*LEPR*	G	0.961	0.807 - 1.145	0.658	0.962	0.803 - 1.154	0.678	0.994	0.948	0.789 - 1.138	0.566	0.987	0.823 - 1.184	0.889	0.757
rs12970134	*MC4R*	A	1.067	0.874 - 1.302	0.524	0.991	0.799 - 1.229	0.934	0.626	1.023	0.830 - 1.260	0.833	1.010	0.814 - 1.253	0.931	0.933
rs1501299	*ADIPOQ*	T	0.915	0.759 - 1.105	0.356	0.935	0.767 - 1.138	0.501	0.881	1.040	0.852 - 1.270	0.698	0.965	0.792 - 1.175	0.722	0.599
rs1558902	*FTO*	A	1.125	0.951 - 1.332	0.170	1.145	0.953 - 1.376	0.148	0.893	1.154	0.966 - 1.379	0.113	1.098	0.913 - 1.320	0.320	0.707
rs16139	*NPY*	C	0.692	0.460 - 1.043	0.079	0.850	0.461 - 1.567	0.602	0.573	0.887	0.570 - 1.381	0.595	0.903	0.489 - 1.667	0.744	0.963
rs17782313	*MC4R*	C	1.069	0.870 - 1.312	0.526	0.993	0.800 - 1.232	0.946	0.630	1.004	0.809 - 1.245	0.974	1.015	0.817 - 1.260	0.894	0.944
rs1801282	*PPARγ*	C	1.088	0.841 - 1.407	0.521	0.730	0.474 - 1.124	0.153	0.134	1.046	0.797 - 1.372	0.748	0.701	0.452 - 1.088	0.113	0.142
rs2241766	*ADIPOQ*	G	0.871	0.664 - 1.143	0.320	0.874	0.660 - 1.157	0.347	0.989	0.902	0.677 - 1.202	0.481	0.940	0.709 - 1.246	0.666	0.842
rs2815752	*NEGR1*	C	1.061	0.885 - 1.272	0.521	1.107	0.904 - 1.355	0.326	0.763	1.016	0.839 - 1.231	0.870	1.073	0.876 - 1.315	0.494	0.706
rs2867125	*TMEM18*	C	1.319	1.065 - 1.633	0.011	1.063	0.825 - 1.369	0.636	0.191	1.367	1.094 - 1.706	0.006	1.046	0.811 - 1.348	0.731	0.111
rs6265	*BDNF*	C	0.988	0.796 - 1.225	0.910	0.893	0.671 - 1.188	0.438	0.575	0.977	0.778 - 1.227	0.842	0.927	0.697 - 1.233	0.601	0.772
rs6499640	*FTO*	A	1.053	0.886 - 1.252	0.556	1.031	0.862 - 1.233	0.737	0.867	1.099	0.916 - 1.319	0.309	1.052	0.880 - 1.258	0.579	0.734
rs6548238	*TMEM18*	C	1.334	1.079 - 1.651	0.008	1.142	0.868 - 1.504	0.343	0.371	1.386	1.111 - 1.730	0.004	1.116	0.848 - 1.469	0.434	0.218
rs659366	*UCP2*	C	0.978	0.816 - 1.171	0.805	1.055	0.874 - 1.272	0.578	0.563	1.029	0.852 - 1.244	0.766	1.102	0.914 - 1.329	0.309	0.612
rs660339	*UCP2*	G	0.994	0.832 - 1.187	0.945	1.053	0.875 - 1.267	0.583	0.654	1.062	0.882 - 1.280	0.524	1.121	0.932 - 1.349	0.227	0.686
rs925946	*BDNF*	T	0.976	0.800 - 1.190	0.807	1.001	0.832 - 1.204	0.990	0.852	0.906	0.736 - 1.116	0.355	1.046	0.869 - 1.258	0.633	0.316
rs9939609	*FTO*	A	1.124	0.950 - 1.330	0.173	1.151	0.959 - 1.382	0.131	0.854	1.158	0.970 - 1.383	0.105	1.138	0.947 - 1.367	0.167	0.894
rs9941349	*FTO*	T	1.167	0.985 - 1.383	0.074	1.207	1.004 - 1.450	0.045	0.797	1.212	1.013 - 1.449	0.035	1.159	0.964 - 1.393	0.116	0.738

‡Difference in association with quantitative traits between study populations. *p≤0.05 and **p≤0.0025.
Results in grey highlights the significant associations between the SNP and BMI by populations.

Figure S1. Participant flowchart for the samples included in the current analysis

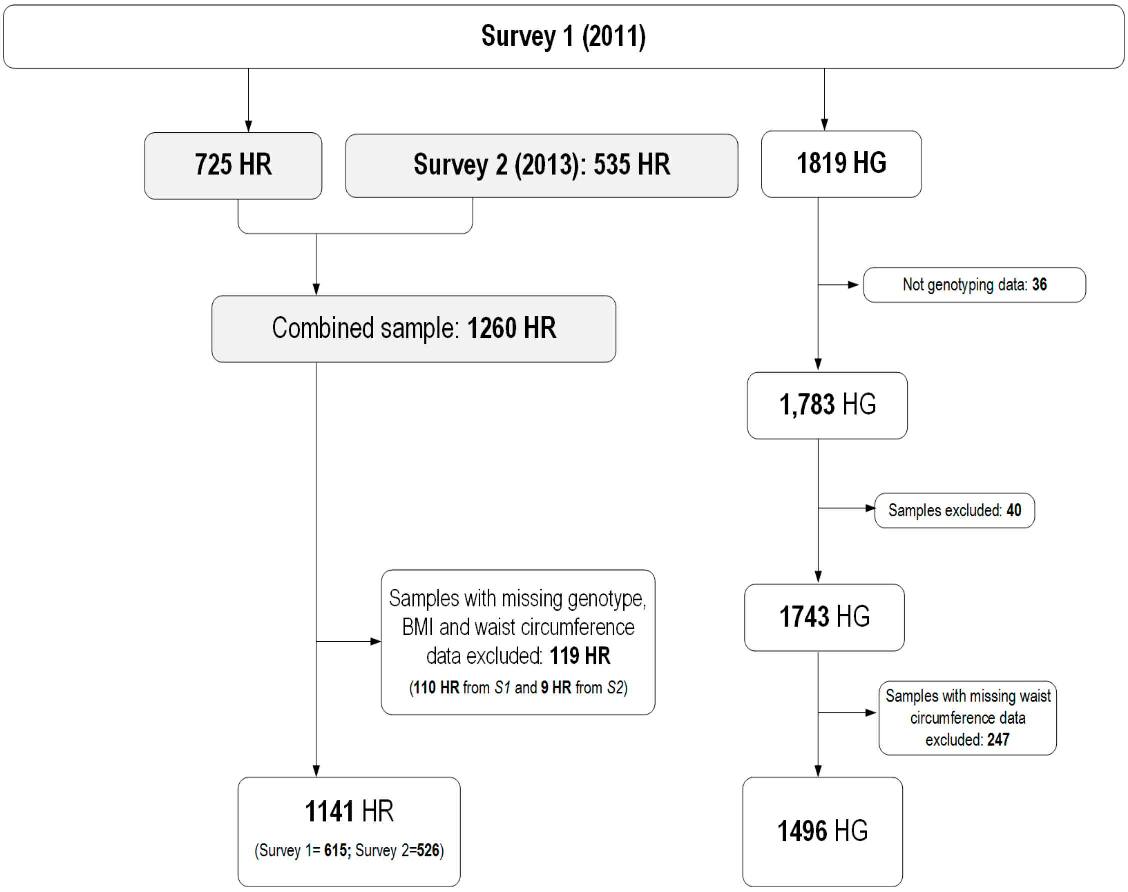

HR: Hungarian Roma subjects; HG: Hungarian general subjects; BMI: Body mass index.

Table S1. Demographic details for Hungarian general and Roma subjects

**Characteristics**	**HG S1 (N= 1496)**	**HR S1 (N= 615)**	**HR S2 (N=526)**
Females (%) / Males (%)	52.87/47.13	61.53/38.47	57.73/42.27
Age (mean value (years) ± SD)	44.17±11.81	40.53±11.37	39.75±14.86

HG S1: Hungarian general subjects from the first survey; HR S1: Hungarian Roma subjects from the first survey; HR S2: Hungarian Roma subjects from the second survey 2; SD: Standart deviation.

Distribution of allele frequencies of individual SNPs and LD analysis in both populations

Ten obesity-related polymorphisms showed significant difference in frequencies of the risk allele between the two study groups (six of them had significantly higher frequency in HR and five in the HG population – see Table 3).

Table S2. Allele frequencies of individual SNPs in the Hungarian general and Roma populations analysed in the current study

Gene	SNP	Risk allele	Risk allele frequency		p-value‡
			HG	HR	
LEPR	rs1137101	G	45.10%	43.92%	0.691
NEGR1	rs2815752	A	65.72%	73.18%	**<0.001****
TMEM18	rs2867125	C	81.41%	85.90%	**<0.001****
	rs6548238	C	81.24%	88.88%	**<0.001****
PPARγ	rs1801282	C	12.63%	4.86%	**<0.001****
ADIPOQ	rs2241766	G	10.98%	10.77%	0.266
	rs1501299	T	28.90%	27.43%	0.286
GNPDA2	rs10938397	G	45.28%	40.90%	**0.008***
NPY	rs16139	C	4.33%	2.27%	**<0.001****
BDNF	rs925946	T	25.41%	37.38%	**<0.001****
	rs6265	C	80.17%	89.21%	**<0.001****
UCP2	rs660339	G	60.18%	63.64%	**0.021***
	rs659366	C	64.12%	65.34%	0.680
FTO	rs6499640	A	58.57%	51.34%	**<0.001****
	rs1558902	A	44.90%	42.46%	0.051
	rs1121980	A	46.44%	46.14%	0.312
	rs9939609	A	43.26%	42.27%	0.178
	rs9941349	T	44.31%	43.10%	0.258
MC4R	rs17782313	C	22.41%	23.00%	0.338
	rs12970134	A	24.48%	23.24%	0.362

HG = Hungarian general population; HR = Hungarian Roma population.
‡p-value for difference in association between study population: * nominal significance and ** multiple comparison adjusted significance level for cross-group comparison.

Four LD blocks were identified in both study populations: LD block1: rs2867125 and rs6548238; LD block2: rs1558902, rs1121980, rs9939609, rs9941349; LD block3: rs17782313 and rs12970134; LD block4: rs660339 and rs659366 (see Figure S4).

Figure S4. Linkage disequilibrium map of SNPs related to obesity for Hungarian general (A) and Roma (B)*

A

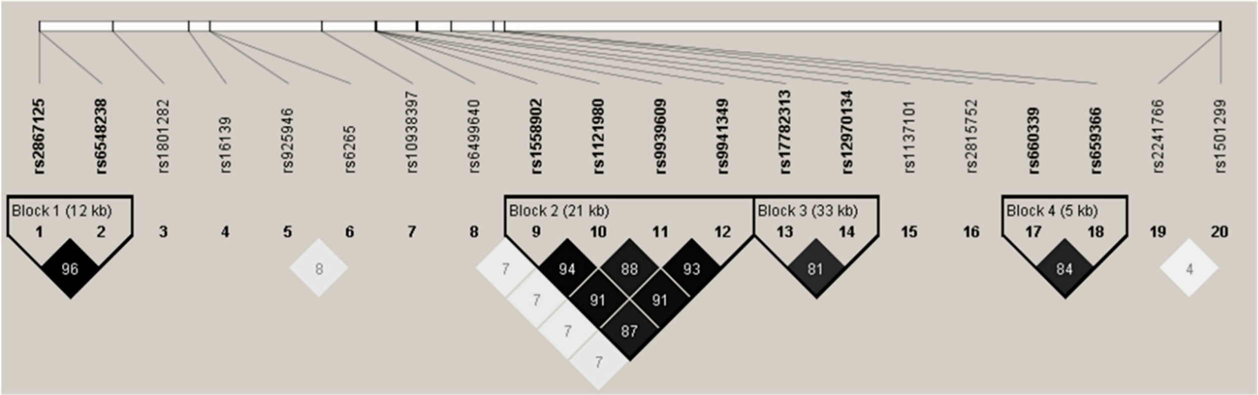

B

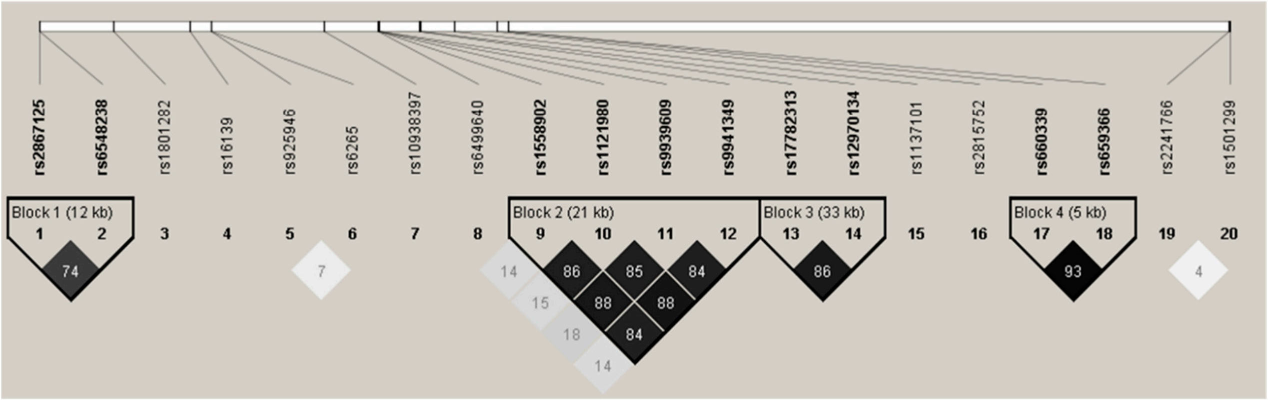

*Linkage analyses were performed separately in the study populations, and 4 blocks were identified. The numbers above the map show the *rs* numbers of SNPs. Alternative R-squared (R^2) colour scheme is used to display LD with (white D'<1 and LOD<2; and black D'<1 and LOD≥2) Numbers in squares are r^2 values.

Figure S2. Summary of SNPs with significant effect on waist circumference with and without significant effect on BMI as continuous outcomes

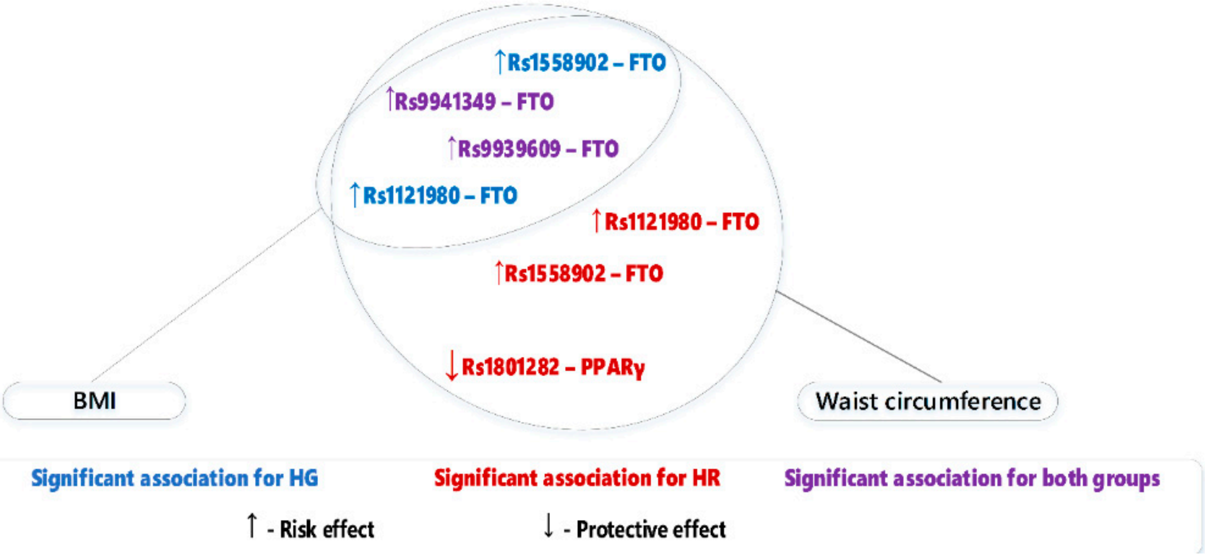

Two SNPs in the *FTO* gene (rs9939609 and rs9941349) were associated with WC and BMI risk in both populations (in purple). Two additional *FTO* SNPs (rs1121980 and rs1558902) were associated with both WC and BMI in the HG population (in blue), but in HR the same two SNPs were associated only with risk for abdominal obesity indicated by increased WC (in red). Rs1801282 (in the *PPARγ* gene) the only distinctive SNP showing a protective effect against increased WC only in HR group.

Figure S3. Summary of SNPs with significant effect on different obesity and abdominal obesity phenotypes

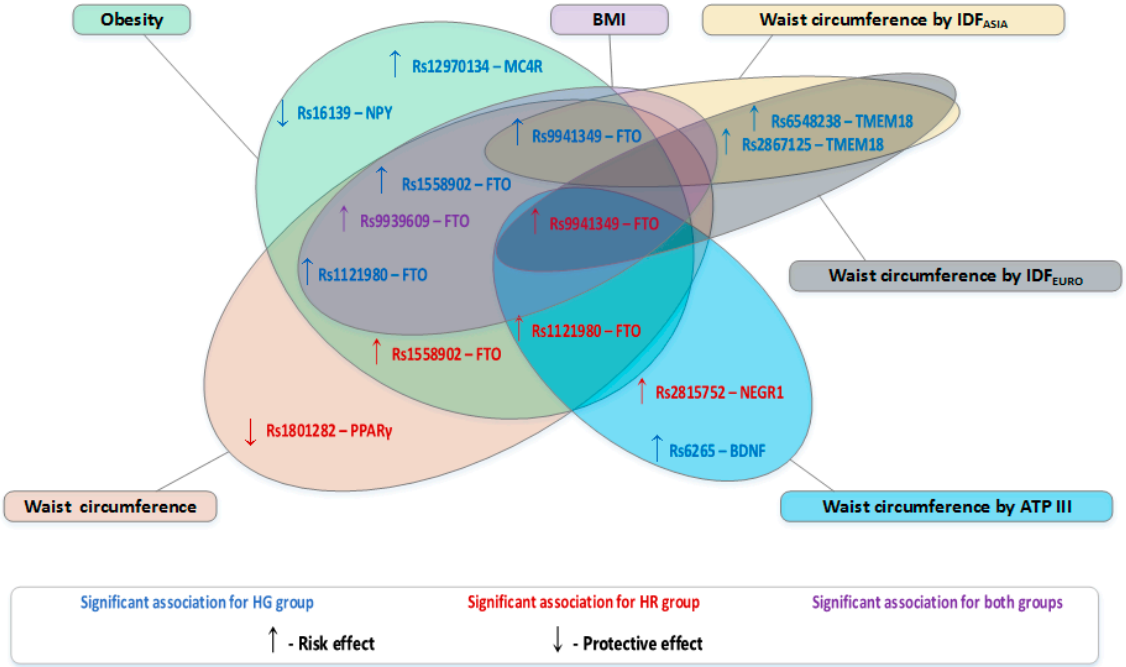

Meta-analysis for PPARγ (rs1801282)

A total of 29 articles were identified for *PPARγ* (rs1801282) and obesity in Asian or Indian populations, by searching 2 databases (Web of science and PubMed) and from additional sources. Potential and relevant titles with summaries were identified in 3 records after removing duplicates. These three full-text articles were further evaluated for eligibility and fulfilled the inclusion criteria. The inclusion criteria for this meta-analysis consisted of the following criteria: (1) study subjects must be from Asian countries, preferably North South India or surrounding countries, (2) study should have been published in peer-reviewed journal with original data, (3) study should investigate the association of *PPARγ* (rs1801282) and obesity-related phenotypes (4) study design should confirm to case vs controls, (5) study should use WHO criteria for obesity and (6) method of genotyping should be explained or linked to a reference. We have excluded studies for (1) overlapping and insufficient data, (2) studies that are conducted with subjects outside North/ South India or surrounding countries and (3) review articles.

Web of science (search string)

(TS=(obesity AND rs1801282 AND Asia*)) AND LANGUAGE: (English) Indexes=SCI-EXPANDED, SSCI, A&HCI, CPCI-S, CPCI-SSH, BKCI-S, BKCI-SSH, ESCI, CCR-EXPANDED, IC Timespan=1975-2020
= 9 results

(TS=(obesity AND rs1801282) AND CU=(india)) AND LANGUAGE: (English) Indexes=SCI-EXPANDED, SSCI, A&HCI, CPCI-S, CPCI-SSH, BKCI-S, BKCI-SSH, ESCI, CCR-EXPANDED, IC Timespan=1975-2020
= 6 results

PubMed (search string)

("obesity"[MeSH Terms] OR "obesity"[All Fields]) AND rs1801282[All Fields] AND ("india"[MeSH Terms] OR "india"[All Fields])
= 3 results

("obesity"[MeSH Terms] OR "obesity"[All Fields]) AND rs1801282[All Fields] AND ("asia"[MeSH Terms] OR "asia"[All Fields])
= 9 results

Additional studies: 2

Figure S5. Flow diagram of study selection for *PPARγ* (rs1801282) polymorphism

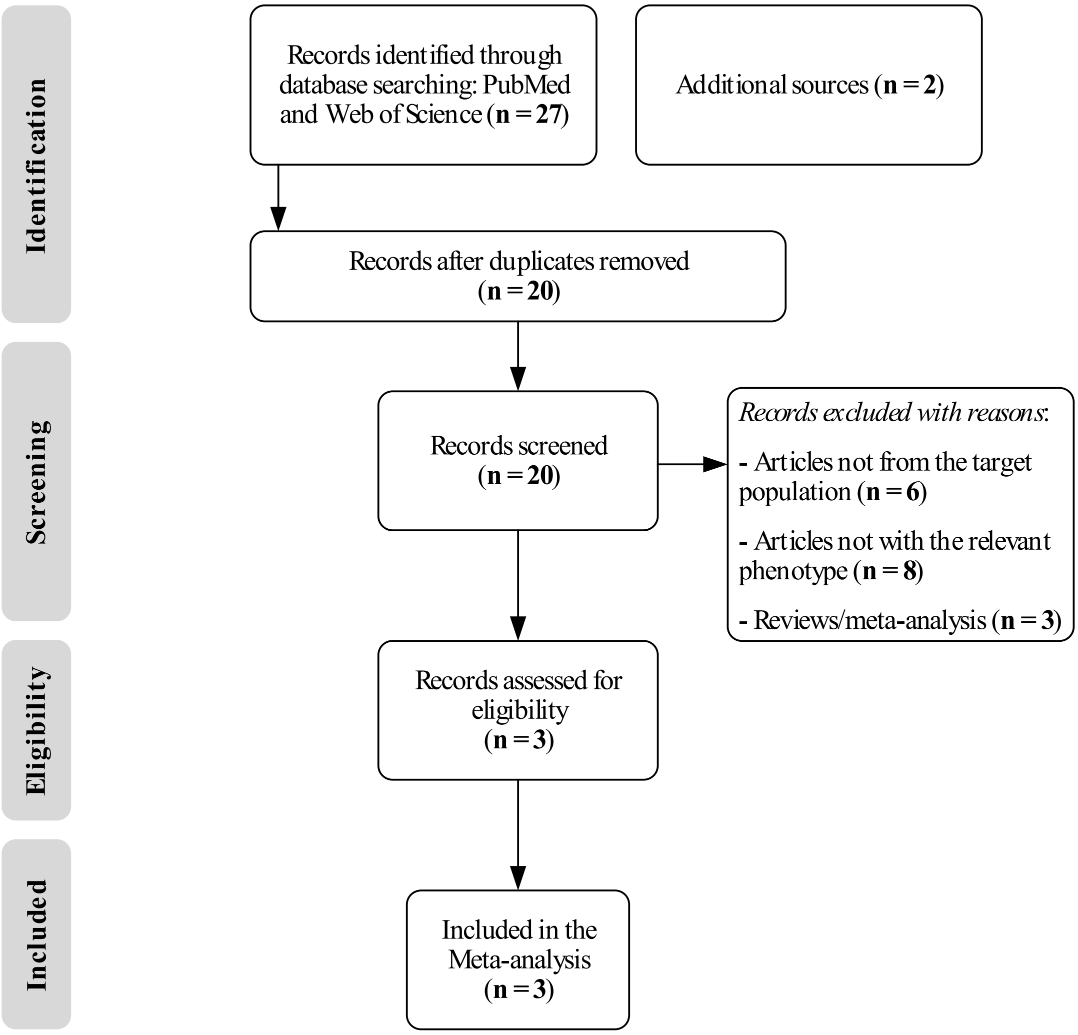

Figure S6. Meta-analysis for studies of *PPARγ* (rs1801282) polymorphism with obesity.

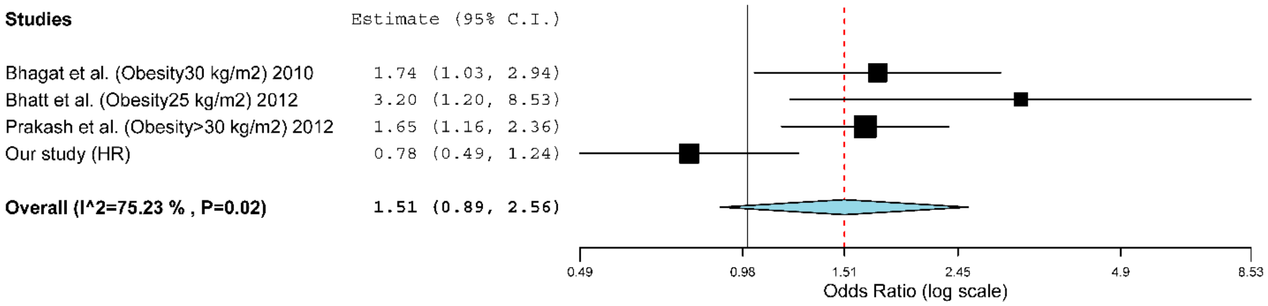

The results of this meta-analysis are insufficient to draw any firm conclusions. However, these results show that HR populations differs from the native Indian population with regards to direction of the association of obesity phenotype and *PPARγ* (rs1801282) SNP.

Table S6. Comparison of adjusted odds ratio of genetic models for *PPARγ* (rs1801282) polymorphism with different phenotyping criteria among HG population

Outcome: Waist circumference ATPIII

Gene (rsID)	Genotype/allele	Case	Control	Allelic OR (95% CI)	Co-dominant OR (95 % CI)	Dominant OR (95 % CI)	Recessive OR (95 % CI)
				G vs C	GG vs GC, CC	GG vs GC+CC	GG+GC vs CC
rs1801282 *PPARγ*	CC	587 (76%)	543 (76.6%)	1.13 (0.89-1.44)	1.00	1.00	1.00
	GC	173 (22.4%)	155 (21.9%)		0.87 (0.66-1.15)	0.87 (0.66-1.13)	0.83 (0.33-2.06)
	GG	12 (1.6%)	11 (1.6%)		0.80 (0.32-2.00)		
	Allele G	197 (12.8%)	177 (12.5%)	p=0.291	p=0.57	p=0.30	p=0.68
	Allele C	1347 (87.2%)	1241 (87.5%)				
	AIC				1770.6	1768.7	1769.6
	BIC				1797.1	1789.9	1790.8

Outcome: Waist circumference IDF EU

Gene (rsID)	Genotype/allele	Case	Control	Allelic OR (95% CI)	Co-dominant OR (95 % CI)	Dominant OR (95 % CI)	Recessive OR (95 % CI)
				G vs C	GG vs GC, CC	GG vs GC+CC	GG+GC vs CC
rs1801282 *PPARγ*	CC	340 (76.6%)	790 (76.2%)	1.09 (0.84-1.41)	1.00	1.00	1.00
	GC	96 (21.6%)	232 (22.4%)		0.97 (0.72-1.29)	0.94 (0.71-1.25)	0.64 (0.25-1.64)
	GG	8 (1.8%)	15 (1.4%)		0.64 (0.25-1.63)		
	Allele G	112 (12.6%)	262 (12.6%)	p=0.52	p=0.64	p=0.66	p=0.36
	Allele C	776 (87.4%)	1812 (87.4%)				
	AIC				1596.2	1594.9	1594.3
	BIC				1622.7	1616.1	1615.5

Outcome: Waist circumference IDF ASIAN

Gene (rsID)	Genotype/allele	Case	Control	Allelic OR (95% CI)	Co-dominant OR (95 % CI)	Dominant OR (95 % CI)	Recessive OR (95 % CI)
				G vs C	GG vs GC, CC	GG vs GC+CC	GG+GC vs CC
rs1801282 *PPARγ*	CC	277 (76.7%)	853 (76.2%)	1.05 (0.80-1.37)	1.00	1.00	1.00
	GC	78 (21.6%)	250 (22.3%)		0.98 (0.72-1.34)	0.97 (0.72-1.31)	0.77 (0.28-2.12)
	GG	6 (1.7%)	17 (1.5%)		0.77 (0.28-2.12)		
	Allele G	90 (12.5%)	284 (12.7%)	p=0.75	p=0.88	p=0.83	p=0.62
	Allele C	632 (87.5)	1956 (87.3%)				
	AIC				1475.5	1473.7	1473.5
	BIC				1502	1494.9	1494.7

Outcome: Obesity (body mass index (BMI) less than 25 kg/m² (as a reference group) vs.BMI≥30 kg/m²

Gene (rsID)	Genotype/allele	Case	Control	Allelic OR (95% CI)	Co-dominant OR (95 % CI)	Dominant OR (95 % CI)	Recessive OR (95 % CI)
				G vs C	GG vs GC, CC	GG vs GC+CC	GG+GC vs CC
rs1801282 *PPARγ*	CC	393 (75.4%)	328 (77%)	1.2 (0.90-1.67)	1.00	1.00	1.00
	GC	120 (23%)	93 (21.8%)		0.81 (0.57-1.14)	0.80 (0.57-1.12)	0.74 (0.21-2.58)
	GG	8 (1.5%)	5 (1.2%)		0.70 (0.20-2.47)		
	Allele G	136 (13.1%)	103 (12.1%)	p=0.194	p=0.43	p=0.20	p=0.63
	Allele C	906 (86.9%)	749 (87.9%)				
	AIC				1129.8	1127.8	1129.2
	BIC				1154	1147.2	1148.6

P < 0.05 and OR with corresponding 95 % CI > 1 are represented in bold

*Akaike information criterion (AIC) and Bayesian information criterion (BIA) for three genetic models. Lower the AIC and BIC value better the model.

Selected genetic model after considering Akaike information criterion for OR (95 % CI) and P value < 0.05 is considered significant, Odds ratio (OR) and corresponding 95 % confidence interval (CI) adjusted for age, sex as covariates.

Table S7. Comparison of adjusted odds ratio of genetic models for *PPARγ* (rs1801282) polymorphism with different phenotyping criteria among HR population

Outcome: Waist circumference ATPIII							
Gene (rsID)	Genotype/allele	Case	Control	Allelic OR (95% CI)	Co-dominant OR (95 % CI)	Dominant OR (95 % CI)	Recessive OR (95 % CI)
				G vs C	GG vs GC, CC	GG vs GC+CC	GG+GC vs CC
rs1801282 *PPARγ*	CC	576 (92%)	378 (88.3%)	0.73 (0.49-1.10)	1.00	1.00	1.00
	GC	49 (7.8%)	48 (11.2%)		1.46 (0.95-2.26)	1.47 (0.96-2.26)	1.60 (0.13-18.98)
	GG	1 (0.2%)	2 (0.5%)		1.66 (0.14-19.65)		
	Allele G	51 (4.1%)	52 (6.1%)	p=0.14	p=0.22	p=0.08	p=0.71
	Allele C	1201 (95.9%)	804 (93.9%				
	AIC				1356	1354	1356.9
	BIC				1380.8	1373.8	1376.7

Outcome: Waist circumference IDF EU							
Gene (rsID)	Genotype/allele	Case	Control	Allelic OR (95% CI)	Co-dominant OR (95 % CI)	Dominant OR (95 % CI)	Recessive OR (95 % CI)
				G vs C	GG vs GC, CC	GG vs GC+CC	GG+GC vs CC
rs1801282 *PPARγ*	CC	421 (92.3%)	533 (89.1%)	0.73 (0.47- 1.12)	1.00	1.00	1.00
	GC	34 (7.5%)	63 (10.5%)		1.43 (0.91-2.25)	1.41 (0.90-2.20)	0.80 (0.07-9.53)
	GG	1 (0.2%)	2 (0.3%)		0.83 (0.07-9.83)		
	Allele G	36 (3.9%)	67 (5.6%)	p=0.15	p=0.28	p=0.13	p=0.86
	Allele C	876 (96.1%)	1129 (94.4%)				
	AIC				1377.1	1375.3	1377.6
	BIC				1401.9	1395.1	1397.5

Outcome: Waist circumference IDF ASIAN							
Gene (rsID)	Genotype/allele	Case	Control	Allelic OR (95% CI)	Co-dominant OR (95 % CI)	Dominant OR (95 % CI)	Recessive OR (95 % CI)
				G vs C	GG vs GC, CC	GG vs GC+CC	GG+GC vs CC
rs1801282 *PPARγ*	CC	394 (92.7%)	560 (89%)	0.70 (0.45-1.09)	1.00	1.00	1.00
	GC	30 (7.1%)	67 (10.7%)		1.50 (0.95-2.38)	1.48 (0.94-2.32)	0.79 (0.07-9.42)
	GG	1 (0.2%)	2 (0.3%)		0.82 (0.07-9.75)		
	Allele G	32 (3.8%)	71 (5.6%)	p=0.11	p=0.21	p=0.088	p=0.85
	Allele C	818 (96.2%)	1187 (94.4%)				
	AIC				1371.9	1370.1	1373
	BIC				1396.7	1390	1392.9

Outcome: Obesity (body mass index (BMI) less than 25 kg/m² (as a reference group) vs.BMI≥30 kg/m²							
Gene (rsID)	Genotype/allele	Case	Control	Allelic OR (95% CI)	Co-dominant OR (95 % CI)	Dominant OR (95 % CI)	Recessive OR (95 % CI)
				G vs C	GG vs GC, CC	GG vs GC+CC	GG+GC vs CC
rs1801282 *PPARγ*	CC	457 (91.6%)	278 (88.5%)	0.78 (0.49-1.24)	1.00	1.00	1.00
	GC	41 (8.2%)	36 (11.5%)		1.36 (0.84-2.19)	1.33 (0.82-2.13)	0.00 (0.00-NA)
	GG	1 (0.2%)	0 (0%)		0.00 (0.00-NA)		
	Allele G	43 (4.3%)	18 (2.9%)	p=0.29	p=0.28	p=0.25	p=0.32
	Allele C	955 (95.7%)	592 (94.3%)				
	AIC				1071.9	1071.1	1071.5
	BIC				1095.4	1089.9	1090.3

*Akaike information criterion (AIC) and Bayesian information criterion (BIA) for three genetic models. Lower the AIC and BIC value better the model.

Selected genetic model after considering Akaike information criterion for OR (95 % CI) and P value < 0.05 is considered significant, Odds ratio (OR) and corresponding 95 % confidence interval (CI) adjusted for age, sex as covariates.

Table S8. Comparison of adjusted odds ratio for a genetic model for candidate gene polymorphism *PPARγ* (rs1801282)

Gene (rsID)	Genotype/ allele	Hungarian general	Hungarian Roma	Allelic OR (95% CI)	Co-dominant OR (95 % CI)	Dominant OR (95 % CI)	Recessive OR (95 % CI)
				G vs C	GG vs GC, CC	GG vs GC+CC	GG+GC vs CC
PPARγ (rs1801282)	CC	1130 (76.3%)	959 (90.6%)	2.74 (2.18-3.46)	1.00	1.00	1.00
	GC	328 (22.1%)	97 (9.2%)		0.36 (0.28-0.46)	0.35 (0.27-0.44)	0.19 (0.06-0.65)
	GG	23 (1.6%)	3 (0.3%)		0.16 (0.05-0.55)		
	Allele G	374 (12.6%)	103 (4.9%)	p<0.001	p<0.0001	p<0.0001	p=0.0014
	Allele C	2588 (87.4%)	2015 (95.1%)				
	AIC*				3297.5	3297.4	3370.8
	BIC*				3326.7	3320.7	3394.2

P < 0.05 and OR with corresponding 95 % CI > 1 are represented in bold

*Akaike information criterion (AIC) and Bayesian information criterion (BIA) for three genetic models. Lower the AIC and BIC value better the model. Selected genetic model after considering Akaike information criterion for OR (95 % CI) and P value < 0.05 is considered significant, Odds ratio (OR) and corresponding 95 % confidence interval (CI) adjusted for age, sex as covariates.

Table S9. Comparison of adjusted odds ratio for a genetic model for the association of the candidate gene polymorphism *PPARγ* (rs1801282) and obesity (adjusted for ethnicity, age and sex)

Gene (rsID)	Genotype/ allele	Case (BMI≥30)	Control (normal BMI	Allelic OR (95% CI)	Co-dominant OR (95 % CI)	Dominant OR (95 % CI)	Recessive OR (95 % CI)
				G vs C	GG vs GC, CC	GG vs GC+CC	GG+GC vs CC
rs1801282 *PPARγ*	CC	850 (83.3%)	606 (81.9%)	1.06 (0.83-1.36)	1.00	1.00	1.00
	GC	161 (15.8%)	129 (17.4%)		0.97 (0.74-1.27)	0.96 (0.73-1.25)	0.68 (0.21-2.13)
	GG	9 (0.9%)	5 (0.7%)		0.67 (0.21-2.12)		
	Allele G	1861 (91.2%)	1341 (90.6%)				
	Allele C	179 (8.8%)	139 (9.4%)	p=0.645	p=0.78	p=0.74	p=0.5
	AIC				2240.7	2239.1	2238.8
	BIC				2273.6	2266.5	2266.1

References

1. Loos RJ, Yeo GS. The bigger picture of FTO - the first GWAS-identified obesity gene. Nature Reviews Endocrinology. 2014;10(1):51-61.
2. Poveda A, Ibáñez ME, Rebato EJAJoHB. Common variants in BDNF, FAIM2, FTO, MC4R, NEGR1, and SH2B1 show association with obesity-related variables in S panish R oma population. 2014;26(5):660-9.
3. Cecil JE, Tavendale R, Watt P, Hetherington MM, Palmer CN. An obesity-associated FTO gene variant and increased energy intake in children. New England Journal of Medicine. 2008;359(24):2558-66.
4. Timpson NJ, Emmett PM, Frayling TM, Rogers I, Hattersley AT, McCarthy MI, et al. The fat mass–and obesity-associated locus and dietary intake in children–. The American journal of clinical nutrition. 2008;88(4):971-8.
5. Wardle J, Carnell S, Haworth CM, Farooqi IS, O'Rahilly S, Plomin R. Obesity associated genetic variation in FTO is associated with diminished satiety. The Journal of Clinical Endocrinology & Metabolism. 2008;93(9):3640-3.
6. Clement K, editor Monogenic forms of obesity: from mice to human. Annales d'endocrinologie; 2000.
7. Fischer J, Koch L, Emmerling C, Vierkotten J, Peters T, Brüning JC, et al. Inactivation of the Fto gene protects from obesity. Nature. 2009;458(7240):894.

8. Cheung M, Gulati P, O'rahilly S, Yeo G. FTO expression is regulated by availability of essential amino acids. International journal of obesity. 2013;37(5):744.
9. Gulati P, Cheung MK, Antrobus R, Church CD, Harding HP, Tung Y-CL, et al. Role for the obesity-related FTO gene in the cellular sensing of amino acids. Proceedings of the National Academy of Sciences. 2013;110(7):2557-62.
10. Gulati P, Yeo GS. The biology of FTO: from nucleic acid demethylase to amino acid sensor. Diabetologia. 2013;56(10):2113-21.
11. Smemo S, Tena JJ, Kim K-H, Gamazon ER, Sakabe NJ, Gómez-Marín C, et al. Obesity-associated variants within FTO form long-range functional connections with IRX3. Nature. 2014;507(7492):371.
12. Mačeková S, Bernasovský I, Gabriková D, Bôžiková A, Bernasovská J, Boroňová I, et al. Association of the FTO rs9939609 polymorphism with obesity in Roma/Gypsy population. 2012;147(1):30-4.
13. Thorleifsson G, Walters GB, Gudbjartsson DF, Steinthorsdottir V, Sulem P, Helgadottir A, et al. Genome-wide association yields new sequence variants at seven loci that associate with measures of obesity. Nature genetics. 2009;41(1):18-24.
14. Almén MS, Jacobsson JA, Shaik JH, Olszewski PK, Cedernaes J, Alsiö J, et al. The obesity gene, TMEM18, is of ancient origin, found in majority of neuronal cells in all major brain regions and associated with obesity in severely obese children. BMC medical genetics. 2010;11(1):58.
15. Loos RJ, Lindgren CM, Li S, Wheeler E, Zhao JH, Prokopenko I, et al. Common variants near MC4R are associated with fat mass, weight and risk of obesity. Nature genetics. 2008;40(6):768.
16. Wang S, Song J, Yang Y, Chawla NV, Ma J, Wang H. Rs12970134 near MC4R is associated with appetite and beverage intake in overweight and obese children: A family-based association study in Chinese population. PLoS ONE. 2017;12(5):e0177983. PubMed PMID: PMC5433775.
17. González-Muniesa P, Mártinez-González M-A, Hu FB, Després J-P, Matsuzawa Y, Loos RJF, et al. Obesity. Nature Reviews Disease Primers. 2017;3:17034. doi: 10.1038/nrdp.2017.34.
18. Magni P, Dozio E, Ruscica M, Celotti F, Masini MA, Prato P, et al. Feeding behavior in mammals including humans. Annals of the New York Academy of Sciences. 2009;1163(1):221-32.
19. Mercer JG, Hoggard N, Williams LM, Lawrence CB, Hannah LT, Morgan PJ, et al. Coexpression of leptin receptor and preproneuropeptide Y mRNA in arcuate nucleus of mouse hypothalamus. Journal of neuroendocrinology. 1996;8(10):733-5.
20. Ahima RS, Prabakaran D, Mantzoros C, Qu D, Lowell B, Maratos-Flier E, et al. Role of leptin in the neuroendocrine response to fasting. Nature. 1996;382(6588):250-2. doi: 10.1038/382250a0.
